# The “Outcome Reporting in Brief Intervention Trials: Alcohol” (ORBITAL) Core Outcome Set: International Consensus on Outcomes to Measure in Efficacy and Effectiveness Trials of Alcohol Brief Interventions

**DOI:** 10.15288/jsad.2021.82.638

**Published:** 2021-09-19

**Authors:** Gillian W. Shorter, Jeremy W. Bray, Nick Heather, Anne H. Berman, Emma L. Giles, Mike Clarke, Carolina Barbosa, Amy J. O'Donnell, Aisha Holloway, Heleen Riper, Jean-Bernard Daeppen, Maristela G. Monteiro, Richard Saitz, Jennifer McNeely, Lela McKnight-Eily, Alex Cowell, Paul Toner, Dorothy Newbury-Birch

**Affiliations:** ^a^ Centre for Improving Health Related Quality of Life, School of Psychology, Queen’s University Belfast, Belfast, United Kingdom; ^b^ Drug and Alcohol Research Network, Queen’s University Belfast, Belfast, United Kingdom; ^c^ School of Social Sciences, Humanities and Law, Teesside University, Middlesbrough, United Kingdom; ^d^ Department of Economics, Bryan School of Business and Economics, University of North Carolina at Greensboro, Greensboro, North Carolina; ^e^ Department of Psychology, Faculty of Health and Life Sciences, Northumbria University, Newcastle upon Tyne, United Kingdom; ^f^ Department of Psychology, Uppsala University, Uppsala, Sweden; ^g^ Stockholm Health Care Services, Stockholm Region, Stockholm, Sweden; ^h^ Department of Clinical Neuroscience, Karolinska Institutet, Stockholm, Sweden; ^i^ School of Health and Life Sciences, Teesside University, Middlesbrough, United Kingdom; ^j^ Northern Ireland Methodology Hub, Queen’s University Belfast, Belfast, United Kingdom; ^k^ Behavioral Health Economics Program, RTI International, Research Triangle Park, North Carolina; ^l^ Population Health Sciences Institute, Newcastle University, Newcastle upon Tyne, United Kingdom; ^m^ Nursing Studies, School of Health in Social Science, University of Edinburgh, Edinburgh, United Kingdom; ^n^ Faculty of Behavioural and Movement Sciences, Clinical Psychology, Vrije Universiteit, Amsterdam, The Netherlands; ^o^ Département universitaire de médecine et santé communautaires (DUMSC), Lausanne University Hospital, Lausanne, Switzerland; ^p^ Pan American Health Organization (PAHO), Washington, DC; ^q^ Department of Community Health Sciences, School of Public Health, Boston University, Boston, Massachusetts; ^r^ Clinical Addiction Research and Education (CARE) Unit, Section of General Internal Medicine, School of Medicine, Boston University, Boston, Massachusetts; ^s^ Grayken Center for Addiction, Boston Medical Center, Boston, Massachusetts; ^t^ New York University Grossman School of Medicine, New York, New York; ^u^ U.S. Centers for Disease Control and Prevention, Atlanta, Georgia

## Abstract

**Objective:**

The purpose of this study was to report the “Outcome Reporting in Brief Intervention Trials: Alcohol” (ORBITAL) recommended core outcome set (COS) to improve efficacy and effectiveness trials/evaluations for alcohol brief interventions (ABIs).

**Method:**

A systematic review identified 2,641 outcomes in 401 ABI articles measured by 1,560 different approaches. These outcomes were classified into outcome categories, and 150 participants from 19 countries participated in a two-round e-Delphi outcome prioritization exercise. This process prioritized 15 of 93 outcome categories for discussion at a consensus meeting of key stakeholders to decide the COS. A psychometric evaluation determined how to measure the outcomes.

**Results:**

Ten outcomes were voted into the COS at the consensus meeting: (a) typical frequency, (b) typical quantity, (c) frequency of heavy episodic drinking, (d) combined consumption measure summarizing alcohol use, (e) hazardous or harmful drinking (average consumption), (f) standard drinks consumed in the past week (recent, current consumption), (g) alcohol-related consequences, (h) alcohol-related injury, (i) use of emergency health care services (impact of alcohol use), and (j) quality of life.

**Conclusions:**

The ORBITAL COS is an international consensus standard for future ABI trials and evaluations. It can improve the synthesis of new findings, reduce redundant/selective reporting (i.e., reporting only some, usually significant outcomes), improve between-study comparisons, and enhance the relevance of trial and evaluation findings to decision makers. The COS is the recommended minimum and does not exclude other, additional outcomes.

Alcohol brief interventions (ABIs) are recommended to help reduce alcohol use among those who are at risk of, or are experiencing, alcohol-related problems but are not seeking treatment (Coffield et al., 2001; National Institute for Health and Clinical Excellence, 2010; Shorter et al., 2020). ABIs can be brief or extended and allow health care staff, laypersons, or other professionals (not part of formal alcohol treatment) to measure and provide feedback on alcohol consumption, offer advice, and provide motivation and support to change drinking behavior.

There is an extensive evidence base on the efficacy of ABIs in primary care settings and online for improving self-reported drinking (Kaner et al., 2018; O’Donnell et al., 2014; Riper et al., 2018). However, evidence for ABIs in other settings is variable (Saitz, 2014). As the ABI field expands using technological advances such as smartphone applications, artificial intelligence, and innovative websites, there is an urgent need to prioritize key outcomes to inform which ABIs are efficacious or effective.

Determining the efficacy/effectiveness of ABIs depends on outcomes selected to identify change (Williamson et al., 2012a, 2017). We have evidence of the diversity of ABI outcome choices currently used in trials, identifying 401 articles reporting 2,641 outcomes measured in 1,560 unique ways (Shorter et al., 2019a). The diversity in outcomes leads to valuable but rarely measured outcomes being missed by meta-analyses (Glasziou, 2014).

The International Network on Brief Interventions for Alcohol and Other Drugs (INEBRIA) organization convened a Research Measurement Standardization Special Interest Group to establish a Core Outcome Set (COS) for ABIs in 2014. The resultant Outcome Reporting in Brief Intervention Trials: Alcohol (ORBITAL) project was tasked with developing this COS. ORBITAL followed Core Outcome Measures in Effectiveness Trials (COMET) guidelines on COS development (Williamson et al., 2012a, 2012b). These guidelines recommend a three-phase process, and Phases 1 and 2 are complete. Phase 1, a systematic review, identified (Phase 1a) and summarized (Phase 1b) outcomes reported in ABI efficacy/effectiveness trials (Shorter et al., 2019a). Phase 2 sought consensus through ranking 84 outcomes in an e-Delphi exercise, based on those identified through the first 100 articles from the Phase 1 review, enhanced with feedback from those with lived experience of unhealthy alcohol use (Shorter et al., 2019b). Outcomes were ranked by 150 e-Delphi participants from 19 countries (nine from lowand middle-income countries), a convenience sample reached through snowball sampling, email lists, known contacts in stakeholder groups, and corresponding authors of ABI evaluations (Shorter et al., 2019b). This article reports Phase 3a, the consensus meeting to discuss and refine outcomes prioritized by the e-Delphi into the final COS; and Phase 3b, selection of instruments based on psychometric properties and suitability to ABIs defined in the scope (Figure 1).

**Figure 1.**
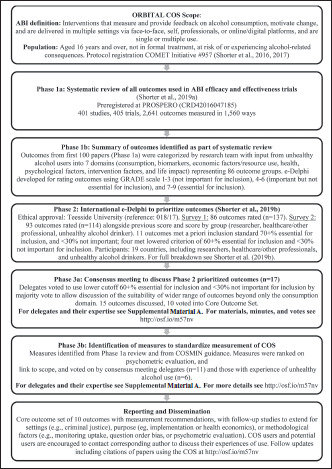
Flowchart of the development of the Outcome Reporting in Brief Intervention Trials for Alcohol (ORBITAL) Core Outcome Set for international efficacy/effectiveness evaluations of Alcohol Brief Interventions

## Method

### Phase 3a study design, participants, and consensus meeting procedure

The consensus meeting was held in New York (United States) on the day before the 14th International INEBRIA conference. Invitations were sent to 31 individuals based on experience in the ABI field, and 17 attended (delegate background, ABI area, and related experience summarized in Supplemental Material A). Those who refused could not attend the day before the conference or were not attending the full INEBRIA conference.

All delegates at the consensus meeting (*n* = 17) had equal voting rights and received a summary of the results of the e-Delphi before the meeting. The meeting opened with the COS scope and procedures. Each outcome was presented, with votes from the e-Delphi displayed by the stakeholder group (those with experience of unhealthy alcohol use, health care or other professionals, or researchers). Delegates discussed each outcome for five minutes, emphasizing why an outcome should be excluded. The discussion focused on reasons to exclude because all outcomes were voted as important for consideration by the e-Delphi panel, and the aim of the consensus meeting was to refine the e-Delphi panel decisions into the minimum for the COS. Each person was limited to one minute of speaking time to allow inclusion of a range of views and concise delivery of arguments. At the end of the discussion of each outcome, delegates voted, with a numeric majority deciding outcome inclusion. Details of the discussions are summarized below, with full details available at http://osf.io/m57nv. This approach was guided by COMET Initiative methodology (Williamson et al., 2012a, 2012b).

To compensate for the absence of individuals with lived experience of unhealthy alcohol use, the chair reminded delegates to refer to the e-Delphi views of those individuals as they voted. After delegates had discussed all outcomes, they were split into two equal groups to identify duplication or redundancy in the outcomes selected. Emails of approach to delegates, materials provided in advance and on the day, votes, discussion summaries, psychometric properties, and rankings are available at http://osf.io/m57nv. For this study, *individuals with unhealthy alcohol use* were defined as those currently consuming more than recommended daily, weekly, or per-occasion amounts.

### Phase 3b study design, participants, and selection of outcome measure procedures

Scales and questions to measure the COS outcomes were identified from Shorter et al. (2019a) and Prinsen et al. (2016) recommended measure repositories. Co-authors/delegates and six individuals with experience of unhealthy alcohol use were invited to review the applicability of measures to the outcome and COS scope, review psychometric properties, and indicate preferred measures. The properties evaluated were applicability to ABI settings; sound psychometric value including content, criterion, structural, and cross-cultural validity; internal consistency; measurement error; sensitivity to change; reliability; hypothesis testing; responsiveness; availability; brevity/ease of administration; and overlap with other COS measures (Mokkink et al., 2010). Initial screening eliminated long instruments (>20 minutes to complete), clinician-delivered instruments that require purchase (limits use in low-resource settings), and redundant questionnaires (>50% of questions did not measure outcome).

## Results

Ten outcomes and recommended measures were selected for the COS. Table 1 shows these outcomes represent domains of average drinking, recent drinking, alcohol-related consequences, and quality of life. The meeting delegates agreed the COS questions should begin with a clear guide to a standard drink in the country to help accurate estimation of alcohol use. ABI evaluations should detail the questions and how they are used (e.g., scale score or binary above/below a cutoff point) with the measure of aggregation described (e.g., mean value or mean individual difference) and the time point (e.g., 3-month post-intervention) and must include summary scores by group and measures of data spread. For a data dictionary, see Supplemental Material B.

**Table 1.**
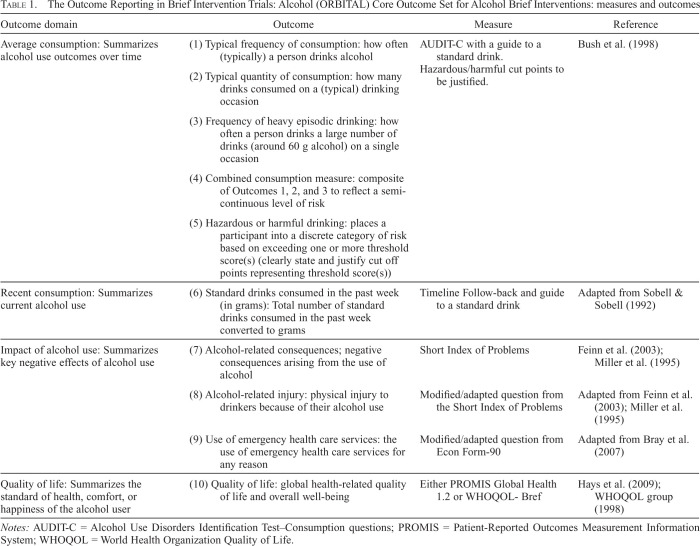
The Outcome Reporting in Brief Intervention Trials: Alcohol (ORBITAL) Core Outcome Set for Alcohol Brief Interventions: measures and outcomes

Outcome domain	Outcome	Measure	Reference
Average consumption: Summarizes alcohol use outcomes over time
	(1) Typical frequency of consumption: how often (typically) a person drinks alcohol	AUDIT-C with a guide to a standard drink. Hazardous/harmful cut points to be justified.	Bush et al. (1998)
	(2) Typical quantity of consumption: how many drinks consumed on a (typical) drinking occasion		
	(3) Frequency of heavy episodic drinking: how often a person drinks a large number of drinks (around 60 g alcohol) on a single occasion		
	(4) Combined consumption measure: composite of Outcomes 1, 2, and 3 to reflect a semi-continuous level of risk		
	(5) Hazardous or harmful drinking: places a participant into a discrete category of risk based on exceeding one or more threshold score(s) (clearly state and justify cut off points representing threshold score(s))		
Recent consumption: Summarizes current alcohol use	(6) Standard drinks consumed in the past week (in grams): Total number of standard drinks consumed in the past week converted to grams	Timeline Follow-back and guide to a standard drink	Adapted from Sobell & Sobell (1992)
Impact of alcohol use: Summarizes key negative effects of alcohol use	(7) Alcohol-related consequences; negative consequences arising from the use of alcohol	Short Index of Problems	Feinn et al. (2003); Miller et al. (1995)
	(8) Alcohol-related injury: physical injury to drinkers because of their alcohol use	Modified/adapted question from the Short Index of Problems	Adapted from Feinn et al. (2003); Miller et al. (1995)
	(9) Use of emergency health care services: the use of emergency health care services for any reason	Modified/adapted question from Econ Form-90	Adapted from Bray et al. (2007)
Quality of life: Summarizes the standard of health, comfort, or happiness of the alcohol user	(10) Quality of life: global health-related quality of life and overall well-being	Either PROMIS Global Health 1.2 or WHOQOL-Bref	Hays et al. (2009); WHOQOL group (1998)

*Notes:* AUDIT-C = Alcohol Use Disorders Identification Test–Consumption questions; PROMIS = Patient-Reported Outcomes Measurement Information System; WHOQOL = World Health Organization Quality of Life.

The first five outcomes are those that describe average drinking levels of a participant, summarizing different elements of drinking in a given period. The sixth outcome can record current, recent drinking behavior with reasonable accuracy. The seventh, eighth, and ninth outcomes covered impact of alcohol use, including a measure of service use, injury, and a summary of measure of consequences. Finally, the last outcome was quality of life, summarizing the standard of health, comfort, or happiness.

### Outcome 1: Typical frequency of consumption

- How often (typically) a person drinks alcohol. Measured by Alcohol Use Disorders Identification Test (AUDIT) Question 1 (Babor et al., 2001; Bush et al., 1998; Saunders et al., 1993).
  Rationale: Discussions centered on this outcome’s importance to illustrate patterns of average consumption and as a component of a composite score (e.g., AUDIT-C). Delegates acknowledged that frequency alone is not meaningful in all settings or populations, with some exceptions including pregnant women, where abstention is recommended, or young adults, where lower typical frequency of consumption may be preferred. Some countries recommend alcohol-free days, and typical drinking measures would capture this. The delegates agreed the period in which individuals describe their typical frequency must be stated in the questionnaire.

### Outcome 2: Typical quantity of consumption

- How many drinks consumed on a (typical) drinking occasion. Measured by AUDIT Question 2 (Babor et al., 2001; Bush et al., 1998; Saunders et al., 1993).
  Rationale: This relates to patterns of average drinking over time, often part of a composite score, and frequently used in ABI meta-analyses. Delegates discussed the potential for subjectivity in determining a typical amount; this may vary by drinking occasion, or there may be no real typical pattern of consumption (e.g., the number of drinks on a Saturday may differ from the number on a weekday). Such inaccuracies may be countered by collecting data on the number of standard drinks consumed in the past week (Outcome 6). The delegates agreed that the period of an individual’s typical quantity must be stated in the questionnaire.

### Outcome 3: Frequency of heavy episodic drinking

- How often a person drinks a large number of drinks (around 60 g alcohol) on a single occasion. Measured by AUDIT Question 3 (Babor et al., 2001; Bush et al., 1998; Saunders et al., 1993).
  Rationale: This item conveys an average of how often individuals drink an excessive number of drinks on a single occasion. The e-Delphi and the consensus delegates viewed this as important. The number of drinks for the setting should approximate to around 60 g of pure alcohol; Babor et al. (2001) provide guidance on how to adjust the question. When calibrating the question, COS users should report grams per drink, which may be four, five, six, or more drinks. Although it may be of less use in ABI trials where abstinence is the behavioral target, it is useful to identify consumption that increases the likelihood of acute consequences. The credibility of an ABI to reduce drinking may be at risk if it did not change this outcome. This outcome can determine a pattern of average drinking with typical frequency and quantity (e.g., AUDIT-C). Delegates agreed the period of an individual’s typical frequency of heavy episodic drinking must be stated in the questionnaire.

### Outcome 4: Combined consumption measure

- Average composite combining Outcomes 1–3 to reflect a semi-continuous level of risk. Measured using the AUDIT-C tool, total score ranging from 0 to 12 (Bush et al., 1998).
  Rationale: Combined consumption measures provide an overall assessment of risk of alcohol-related harm. Delegates thought it impossible to determine what to measure without considering how to measure this outcome. There was a strong preference for the AUDIT-C, given international translation and use, noting the importance of adjusting for country-specific drink size in grams (Babor & Robaina, 2016). There was consideration of whether it was necessary to record the combined measure in every trial, since acute consumption was indicated by the quantity on a single day, and chronic by the frequency. The utility of the combined measure as a summary of risk outweighed this concern.

### Outcome 5: Hazardous or harmful drinking

- Places a participant into a discrete category of risk based on exceeding one or more threshold score(s). Measured using the AUDIT-C; cut-off points will vary by country and setting; clearly state and justify cutoff points representing threshold score(s) (Bush et al., 1998). Rationale: Reducing risk or harm below a pre-determined threshold is often a primary aim of an ABI, with important implications. Although definitions of *hazardous drinking* or* harmful drinking* differ by culture and setting, clearly defined cutoff points, tailored to ABI design, allow meaningful comparisons across evaluations. All individuals with experience of unhealthy alcohol use in the e-Delphi prioritized this outcome, illustrating its importance to consensus meeting delegates. Some argued that risk was better communicated in the ABI feedback element to drinkers themselves, but policy makers also benefit from knowing whether an intervention reduces the risk of harm. Typically, this outcome will be a justified cutoff point for hazardous drinking, although there may be instances in which ABI researchers may wish to include harmful drinking. We note cut-off points may be determined using country-specific guidelines (for an example, see Higgins-Biddle & Babor, 2018).
  For Outcomes 1–5, the original AUDIT from which the AUDIT-C questions are derived typically captures a past-year period. A 1-year period is likely unsuitable to capture change following ABIs, most often evaluated in 3-month waves of data collection (Shorter et al., 2019a). To facilitate evidence synthesis, delegates agreed that the period in which these average measures are obtained must be specified in reports.

### Outcome 6: Standard drinks consumed in the past week

- Total number of standard drinks consumed in the past week converted to grams. Measured using a calendar-method recall question adapted from Sobell and Sobell (1992), asking for the exact number of drinks on every day of the past week using the same country-specific standard drink guide as the AUDIT-C.
  Rationale: This is a frequently measured outcome in ABI trials and a measure of current drinking to a reasonable accuracy standard. Consensus meeting delegates noted the need for daily estimates, since weekly totals in grams do not distinguish between whether someone drinks one drink per day or seven on a single day. There is considerable variability in how this is measured (past week, average week in varied periods, average measure composites, etc.). For utility in future meta-analyses, weekly drinks can be converted to grams to facilitate international comparison, but when presented they should be described as “drinks,” based on the guide provided.

### Outcome 7: Alcohol-related consequences

- Alcohol-related consequences measured by the Short Index of Problems (SIP; Feinn et al., 2003; Miller et al., 1995), using the four-point Likert response categories.
  Rationale: A key motivator to use ABI is to reduce alcohol-related consequences. Given that the target population for ABIs are those who are experiencing or are at risk of harm, some assessment of alcohol-related consequences was considered important. Delegates recognized variation in the interpretation of harm attributed to alcohol by individuals and across countries; this could be a source of error in any questionnaire selected. There was some discussion on whether specific problems, some easily attributed to alcohol, may be more useful, but the e-Delphi participants did not prioritize individual problems. Twelve questionnaires were considered for this outcome. Many were discounted on grounds of validity, as they approximated to diagnoses of dependence. The highest ranked questionnaire was the SIP. This was particularly valued by individuals with experience of unhealthy alcohol use. It also enabled a more nuanced and less time-critical reflection on alcohol-related consequences. For example, scaling the last two questions of the AUDIT questionnaire to time frames of ABI evaluation—for example, a 3-month follow-up—is problematic, because they measure past events not sensitive to change. One person with lived experience of unhealthy alcohol use said it was impossible to make positive progress if you had ever been advised to cut down or had an injury. Another person suggested past consequences indicated by “ever” on these questions can never be solved by a current ABI, because if something had “ever” happened before that ABI it would still be something to report as “ever” happening after the ABI.

### Outcome 8: Alcohol-related injury

- Physical injury to drinkers because of their alcohol use. Single question adapted from SIP (Feinn et al., 2003).
  Rationale: This outcome was important because it is proximal to the drinking occasion and, in some settings, is common. Although there are variations in settings, by country, and there may be limited change over follow-up periods, this is a key alcohol consequence. Clinical audits, chart reviews, questionnaires, and single questions have been used to measure this outcome (Shorter et al., 2019a). Single questions (e.g., the SIP accident question) were considered, but the use of “accidents” can introduce error in attribution of alcohol to measurement of injury (Bonilla-Escobar & Gutiérrez, 2014). Similarly, AUDIT Question 9 was discounted because it refers to harm to self and to others (the latter not voted into the COS), and the scoring of this item limits its sensitivity to change arising from the ABI (because if respondents score yes [2 or 4], they can never score zero in subsequent measurements). An adapted question from the SIP was voted for inclusion.

### Outcome 9: Use of emergency health care services

- Use of emergency health care services for any reason. Question adapted from Econ Form-90 (Bray et al., 2007). If emergency health care is a primary focus, we recommend a follow-up question asking about alcohol-, substance-, or mental health–related visits.
  Rationale: Prevalence of emergency health care use varies by country and ABI setting. There was a concern over the lack of health care measures; health care savings are an often-cited policy reason for adopting ABIs. Emergency care is expensive and is proximally associated with unhealthy alcohol use. Concerns were raised about measurement and whether self-report was valid and reliable. Checking clinical records was too burdensome/costly to recommend for all evaluations. Two measurement approaches were identified: general emergency health care use and use specific to alcohol (Shorter et al., 2019a). General emergency health care use was thought more useful because of difficulties in attributing the reason for attendance to alcohol.

### Outcome 10: Quality of life

- Global health-related quality of life and overall well-being. Two measures are recommended, the WHOQOLBREF (WHOQOL Group, 1998) and the PROMIS Global Health 1.2 (Hays et al., 2009).
  Rationale: ABI beneficiaries care about life quality and not just longevity; this outcome was rated highly in the e-Delphi by ABI beneficiaries, and some consensus meeting delegates felt that an ABI may not be considered useful if it did not influence quality of life. The type of ABI was thought to be relevant. ABIs involving brief counseling will likely address several issues alongside alcohol. As such, issues beyond the ABI might influence quality of life. Most ABI trials use the EQ-5D to measure quality of life. However, although initially favored by consensus delegates, the published trials (25/26) in the systematic review found no significant or clinically relevant differences reported using this measure (Shorter et al., 2019a). Another popular measure is SF-12, but its use incurs a cost. Those with experience of unhealthy alcohol use questioned whether EQ-5D measures could capture relevant change resulting from reduced alcohol use. The recommended instruments were the WHOQOL-BREF or the PROMIS Global Health. The WHOQOL-BREF is longer but more established. To the best of our knowledge, there is no use of the PROMIS Global Health in the ABI field, but it was highly recommended by those delegates with experience in unhealthy alcohol use and has strong psychometric properties.

## Discussion

ABIs are a support to alcohol use change and can reduce the impact of unhealthy alcohol use on morbidity and mortality worldwide. The evidence to identify which ABIs are effacious/effective is compromised by the variability in the conduct and reporting of trials and other evaluations (Shorter et al., 2019a). Use of the ORBITAL COS will enable future research to be directly comparable in systematic reviews and meta-analyses and will provide more rigorous evidence of the efficacy/effectiveness of ABIs. This can support future decision making by policy makers and practitioners based on interventions that show meaningful, consistent change. Although the 10 outcomes should be measured and reported in new trials and evaluations, other outcomes may be measured to supplement this COS, including different types of measures such as fidelity, process, or implementation outcomes. Measure substitution in the ORBITAL COS is strongly discouraged; this will lead to a continuation of the problems with evidence synthesis and selective reporting. The 10 COS outcomes are not all primary outcomes. Trialists need to specify a primary outcome a priori for power and sample size calculations, to register their trial, and to avoid type I error (Freemantle, 2001).

Development of the ORBITAL COS followed guidance from the COMET initiative (Williamson et al., 2017) and was reported using COS-STAR reporting guidelines (Supplemental Material C and D). We pre-registered and published our protocol (Shorter et al., 2016, 2017) and engaged stake-holders, including those with lived experience of unhealthy alcohol use, in decision making. The ORBITAL COS covers domains of average consumption, recent consumption, alcohol-related consequences, and quality of life, which can be supplemented with other outcomes to meet specific trial and methodological objectives. It is suitable for trials and evaluations of ABIs in all settings in which the population is age 16 years or older, drinking at a level causing harm or at risk of harm, and not seeking formal alcohol treatment (National Institute for Health and Clinical Excellence, 2010).

All COS outcomes are self-report measures. This may be because knowledge is lacking regarding widely available objective measures, because there are concerns regarding the sensitivity of objective measures to identify unhealthy alcohol use—particularly at lower levels of consumption—and because of concerns about the relatively high cost of objective measures. However, it is important to avoid social desirability bias in trials where one group gets intensive advice to cut down and then is asked by researchers to self-report if they have cut down (McCam-bridge & Saitz, 2017). Blinding of outcome assessors, confidentiality assurances, and agnostic views on amount of alcohol consumed go some way to overcome self-report biases. Biomarkers, ecological momentary assessment, and transdermal alcohol evaluation are generally desirable (Morgenstern et al., 2014; Van Egmond et al., 2020), but for practical reasons of cost and equipment access they are not recommended in this COS.

Similarly, we would have preferred wider representation from low- and middle-income countries at the consensus meeting and in the e-Delphi. Although the final COS outcomes were all ranked 7–9 (important for inclusion) by more than 70% of those in low- and middle-income countries in the e-Delphi (Shorter et al., 2019b), future validation work is needed to establish the utility of the COS in settings where health care resources may be more limited (Tiburcio Sainz et al., 2020). It was not possible to secure the participation of delegates with lived experience of unhealthy alcohol use in the consensus meeting. However, as we decided at the consensus meeting, extra emphasis was placed on the e-Delphi votes of such participants to ensure that we took their perspectives into account in decisions.

The COS is balanced to reduce respondent burden, maximize follow-up, reflect current thinking on ABIs, and demonstrate good psychometric properties. The first five ORBITAL COS outcomes are measured with three questions (AUDIT-C). Our measure of alcohol-related consequences, the SIP, requires 15 questions to measure a single outcome (Feinn et al., 2003). Using short, well-developed measures, such as PROMIS Alcohol Use (Pilkonis et al., 2016) and the Dutch Problem Index (Cornel et al., 1994), can be explored alongside the SIP, where there is capacity to do so, as these instruments may ultimately reduce respondent burden. We note that the 3-month reference period was most commonly used in the ABI field at present (Shorter et al., 2019a), but this may not be suitable for all studies. However, if time periods are amended from the validated version (e.g., a 3-month reference period in the SIP), we strongly recommend that the time reference change is clearly described and justified. Meta-analyses may consider including sensitivity analyses on this indicator, depending on the research question of the review.

Quality of life would benefit from more psychometric evaluation. Although the EQ-5D is commonly used in health intervention studies (with even small changes useful for health economic evaluation), it has ceiling effects that are less sensitive to change in milder health conditions and may not result in meaningful change for those with unhealthy alcohol use (Shorter et al., 2019a). With no clear alternative to the EQ-5D identified, we recommended two quality-of-life measures, either the WHOQOL-BREF or the PROMIS Global Health 1.2. As researchers conducting ABI evaluations adopt this COS and report findings, an evidence base may emerge to support one over the other. Neither measure has a current associated set of preference weights to support economic analyses; however, PROMIS Global Health can approximate to EQ-5D-3L (see Revicki et al., 2009).

This COS is the product of compromise. Combining every outcome preference into a single, acceptable set of measures that is feasible for use in ABI evaluations is impossible. However, the ORBITAL COS is novel in our field, and its adoption in future ABI trials and evaluations represents an important step change in standardizing outcome reporting and improving the evidence base. Looking forward, ORBITAL COS uptake will be observed and documented. COS adopters are encouraged to contact the corresponding author directly to share successes and challenges of using the COS; this feedback will ensure continued utility and inform future revisions (Williamson et al., 2012a, 2017). We encourage emails around further advances. For example, ORBITAL has inspired additional innovation, including exploration of order effects (Bendtsen et al., 2020) or extensions (e.g., for implementation or economic evaluation). As countries recommend ABIs to address unhealthy alcohol use, the international consensus standardization of the ORBITAL COS should positively impact health worldwide through improved evidence and evaluation practice.

## Acknowledgments

The authors thank the INEBRIA Research Measurement Standardization Special Interest Group and participants at a workshop led by Professors Nick Heather and Jim McCambridge in Warsaw in 2014. We also thank Dr. Liz Gargon, Dr. Sarah Gorst, and Professor Paula Williamson for methodological advice on Core Outcome Sets and Dr. Sophie Chung from ICHOM for advice on psychometric presentation. We thank Nicole Ferguson for help with graphics. We thank Professor Tom Babor for valuable insights at the last review of the Core Outcome Set Outcomes at the end of the consensus meeting. Similarly, we thank Peggy Murray for her contribution as a delegate; she has since retired from the National Institute on Alcohol Abuse and Alcoholism. We invited all delegates at the consensus meeting to co-author this article, and all but two agreed.

Registration: This work is pre-registered at the COMET Initiative (Shorter et al., 2016), and the protocol is freely available at Shorter et al. (2017). Supplemental material for all steps of COS development is available on the Open Science Framework DOI: osf.io/m57nv. Items specifically relating to this manuscript include the invitation email (for consensus delegates; https://osf.io/5jv97), materials provided in advance of the consensus meeting (https://osf.io/wyust), presentation at the consensus meeting (https://osf.io/5jc9d), minutes of the consensus meeting with votes (https://osf.io/n2hyr), the email inviting psychometric review (https://osf.io/vqkp3), psychometric presentation for consensus delegates (https://osf.io/u5j37), psychometric presentation for those with experience of unhealthy alcohol use (https://osf.io/6apmb), the psychometric summary of votes and discussions (https://osf.io/6pt9b), and the diagram summarizing final decisions (https://osf.io/84pes).

## Conflict-of-Interest Statement

Gillian W. Shorter, Jeremy W. Bray, Nick Heather, Emma L. Giles, Carolina Barbosa, Amy J. O’Donnell, Aisha Holloway, Alex Cowell, Paul Toner, Heleen Riper, Jean-Bernard Daeppen, Maristela G. Monteiro, Jennifer Mc-Neely, and Lela McKnight-Eily have no conflict of interests to declare. Anne H. Berman is the author of Swedish-language manuals in book form for the AUDIT and DUDIT as well as the Alcohol-E and DUDIT-E, questionnaires recommended for national use in health care and social services in Sweden. Maristela G. Monteiro has been involved in the development of the AUDIT questionnaire. Mike Clarke is a member of the COMET management group. Dorothy Newbury-Birch is co-president of INEBRIA. Richard Saitz is principal investigator of a study funded by the National Institutes of Health in an award to Boston University, which received medication from Alkermes for that trial.
